# RBM39 Alters Phosphorylation of c-Jun and Binds to Viral RNA to Promote PRRSV Proliferation

**DOI:** 10.3389/fimmu.2021.664417

**Published:** 2021-05-17

**Authors:** Yinna Song, Yanyu Guo, Xiaoyang Li, Ruiqi Sun, Min Zhu, Jingxuan Shi, Zheng Tan, Lilin Zhang, Jinhai Huang

**Affiliations:** ^1^ School of Life Sciences, Tianjin University, Tianjin, China; ^2^ Tianjin Key Laboratory of Function and Application of Biological Macromolecular Structures, Tianjin University, Tianjin, China

**Keywords:** RBM39, c-Jun, porcine reproductive and respiratory syndrome virus, AP-1, phosphorylation, interferon

## Abstract

As transcriptional co-activator of AP-1/Jun, estrogen receptors and NF-κB, nuclear protein RBM39 also involves precursor mRNA (pre-mRNA) splicing. Porcine reproductive and respiratory syndrome virus (PRRSV) causes sow reproductive disorders and piglet respiratory diseases, which resulted in serious economic losses worldwide. In this study, the up-regulated expression of RBM39 and down-regulated of inflammatory cytokines (IFN-β, TNFα, NF-κB, IL-1β, IL-6) were determined in PRRSV-infected 3D4/21 cells, and accompanied with the PRRSV proliferation. The roles of RBM39 altering phosphorylation of c-Jun to inhibit the AP-1 pathway to promote PRRSV proliferation were further verified. In addition, the nucleocytoplasmic translocation of RBM39 and c-Jun from the nucleus to cytoplasm was enhanced in PRRSV-infected cells. The three RRM domain of RBM39 are crucial to support the proliferation of PRRSV. Several PRRSV RNA (nsp4, nsp5, nsp7, nsp10-12, M and N) binding with RBM39 were determined, which may also contribute to the PRRSV proliferation. Our results revealed a complex mechanism of RBM39 by altering c-Jun phosphorylation and nucleocytoplasmic translocation, and regulating binding of RBM39 with viral RNA to prompt PRRSV proliferation. The results provide new viewpoints to understand the immune escape mechanism of PRRSV infection.

## Introduction

RNA-binding proteins (RBPs) have been found in all living organisms (1). Through binding RNAs, RBPs assemble in ribonucleoprotein complexes, which dictate the fate and the function of virtually every cellular RNA molecules. RBPs can bind single-stranded or double-stranded RNAs, and play an important role in regulating RNA metabolism and gene expression as post-transcriptional regulators (2, 3). RBPs are involved in biological processes such as RNA transcription, editing, splicing, transportation and positioning, stability and translation (4). With the extensive reports on the post-transcriptional regulatory mechanism of RBPs, more and more scholars are focusing on their complicated functions. The target RNA of RBPs is variable and diverse, and they have ability to bind to different region of mRNA [such as exons, introns, untranslated regions (UTRs)], or interact with other types of RNA, including non-coding RNAs (5), microRNAs, small interference RNAs (siRNA), t-RNAs, small nucleolar RNA (snoR-NA), telomerase RNA, conjugant small nuclear RNA (snRNA) and the RNA part of signal recognition particles (SRP RNA or 7SL RNA (6–8). These non-coding RNAs form a wide range of secondary structures, which combine with RBP and regulate processes such as RNA splicing, RNA modification (9), protein localization, translation, and maintenance of chromosome stability (10, 11). The functional effects of conventional RBPs depend on the target RNA-RNP complex formation. Simultaneously, the RNP complex helps RNA processing, translation, export and localization (10, 12).

RBM39 (RNA binding motif protein 39), also called CAPER, HCC1.3/1.4,Caperα,FSAP59,RNPC2 (13), is a nuclear protein that is involved in precursor mRNA (pre-mRNA) splicing (14). RBM39 was first identified as an auto-antigen in a hepatocellular carcinoma patient (15). In addition, RBM39 is a transcriptional co-activator of activating protein-1 (AP-1/Jun), estrogen receptors (eg, steroid nuclear receptors ESR1/ER-alpha and ESR2/ER-β) and NF-κB (14, 16). Based on the previous research, the function of RBM39 has been associated with malignant progression in a number of cancers (15). Previous studies have reported RBM39 as a proto-oncogene with important roles in the development and progression of multiple types of malignancies (17). RBM39 is relevant to numerous precursor messenger RNA (pre-mRNA) splicing factors and provide regulating action of a quantity of signal pathway (15, 18). Degradation of RBM39 led to aberrant pre-mRNA splicing, including intron retention and exon skipping in hundreds of gene (15). RBM39 has three RNA recognition motif (RRM) and the C-terminal of RRM3 domain belongs to the U2AF homology motif family (UHM), which mediate protein-protein interactions through a short tryptophan-containing peptide known as the UHM-ligand motif (ULM) (14, 16). However, RBM39 impacting virus proliferation and its role in innate immunity are largely unknown.

The activation protein-1 (AP-1) transcription factors are immediate early response genes involved in a diverse set of transcriptional regulatory processes (19, 20). The AP-1 complex is mainly composed of a Fos family member and a Jun family member encoded by the proto-oncogene, which bind to the DNA target sequence in the form of a homologous or heterodimer complex to regulate the expression of the target gene (21, 22). The AP-1 complex consists of Fos family members, Jun family members, activating transcription factor (ATF) and musculoaponeurotic fibrosarcoma (MAF) (21). The Fos family contains c-Fos, v-Fos, Fos-B, Fra-1 and Fra-2 (21); the Jun family is composed of c-Jun, v-Jun, Jun-B, and Jun-D (19); ATF includes ATF2, ATF/LRF1, B-ATF, JDP1 and JDP2 (23); and MAF comprises C-Maf, MafB, MafA, MafG/F/K and Nrl (23). The c-Jun is a central component of all AP-1 complexes and members of the basic region-leucine zipper (bZIP) family of sequence-specific dimeric DNA-binding proteins (19, 24). In addition, the c-Jun is a transcription factor that recognizes the AP-1 complex and binds to the enhancer heptamer motif 5’-TGA[CG]TCA-3’ (termed AP-1 sites) found in a variety of promoters (14, 19). The c-Jun gene is expressed in multiple cell at low levels, and its expression is in elevated response to many stimuli, including growth factors, cytokines, foreign materials and viral infection (25). Certain stimuli of cells result in activation of the JNK (c-Jun N-terminal kinase) and p38 groups of MAPKs, followed by the c-Jun were phosphorylated by JNKs at two positive regulatory sites (serine 63 and 73) residing within its amino-terminal activation domain (9, 19).

Porcine reproductive and respiratory syndrome virus (PRRSV) is a critical pathogen in pig and can trigger a serious negative impact on the economic development of pigs (26, 27), which mainly causes sow reproductive disorders and piglets respiratory diseases, thus resulting in serious economic losses in the world (28). It is a variety of capsule single-stranded positive-chain virus of arteritis virus (29). Infections by PRRSV often result in increased expression of multiple genes and delayed low-level induction of antiviral cytokines, thereby destroying the early endogenous immune response (30, 31). The 15kb PRRSV genome is expressed through a set of subgenomic mRNA transcripts, each used for the translation of one or two open reading frames (ORFs) (32, 33). The full-length viral RNA encodes two large nonstructural polyproteins, pp1a and pp1ab (34), which are processed by viral proteases to release 14 non-structural proteins (35), including four proteases (nsp1α, nsp1β, nsp2 and nsp4), the RNA-dependent RNA polymerase (nsp9), a helicase (nsp10) and an endonuclease (nsp11) (33, 36). In this study, the RBM39 participated in PRRSV RNA binding and viral proliferation was investigated. The dual regulation effects of RBM39 compete with c-Jun to down-regulated IFN-β production and prompt PRRSV proliferation by stabilizing and binding with viral RNA were illustrated.

## Materials and Methods

### Cell and Virus Cultures

Human embryonic kidney 293 T cells (HEK293 T) and HeLa cells were cultured in Dulbecco’s modified essential medium (DMEM), porcine alveolar macrophages (PAM) cell and PAM cell line 3D4/21 in RPMI-1640 medium (Biological Industries) in incubator at 37°C with 5% CO_2_. All media were obtained from Biological Industries and supplemented with 10% fetal bovine serum (FBS), 100 U/ml penicillin, and 100 µg/ml streptomycin. The PRRSV was preserved in our laboratory and produced by transfection of 3D4/21 cells with the PRRSV-JXwn06 infectious clone plasmid. In this study, the PRRSV was used with a titer of 10^4^ PFU/ml. Sendai virus (SeV) and vesicular stomatitis virus (VSV) was preserved in our laboratory.

### Antibodies and Reagents

Murine RBM39 pAb was prepared by immunizing mice with the purified protein. Anti-PRRSV nsp2 antibody was a gift from Prof. Jun Han of China Agricultural University and Rabbit anti-PRRSV-N protein pAb was purchased from YBio Technology (YB-23941R). Mouse anti-Flag/β-actin/GADPH mAb and Peroxidase-Conjugated Goat anti-Rabbit/Mouse IgG (H+L) were purchased from Yeasen Technology. Rabbit anti-phospho-JNK1+2+3 (Thr183+Tyr185) pAb was purchased from Bioss (bs-1640R); Rabbit anti-c-Jun mAb was purchased from Abways Technology (CY5290); Rabbit anti-phospho-c-Jun (Ser73) mAb was purchased from Cell Signaling Technology (3270T); Rabbit anti-HA pAb, Goat anti-Mouse IgG (H+L) Alexa Fluor 555 and Goat anti-Mouse/Rabbit IgG (H+L) FITC was purchased from Invitrogen; Mouse anti-Flag/HA-tags beads was purchased from Abmart. Phosphatase inhibitors were purchased from APE×BIO. Quick CIP were purchased from Biolabs.

### Plasmids

RBM39 and c-Jun gene was cloned from 3D4/21 cells cDNA as template. Flag/HA-RBM39 and Flag/HA-c-Jun plasmids were constructed by seamless cloning technology. Flag-RRM1, RRM2, RRM3, ΔRRM1, ΔRRM2, ΔRRM3, ΔRRM, ΔNLS plasmids were constructed by reverse amplification by Flag-RBM39 plasmid as template. Plasmid pET-28a-RBM39-X7 that delete the complete 5’-terminal NLS sequence was also constructed by seamless cloning method, to purify the RBM39 protein and make its antibodies. AP-1/IFN-β/Renilla luciferase reporter plasmids were constructed as previously described (29). The primers used above are shown in **Table 1**.

**Table 1:** Primers used in PCR amplification.

Primer name	Primer sequence (5’–3’)
P-RBM39	F: TCTTTCCGAACCCAAGCAC
	R: TCATCGTCTACTTGGAACCAG
P-RBM39-X7	F: CCATTCAGAAAAGACAAGAGC
	R: TCATCGTCTACTTGGAACCAG
P-c-Jun	F: GGATCCATGACTGCAAAGATGGAAACGACCTTCTAC
	R: GGATCCTCAAAACGTTTGCAACTGCTGCGTTAG
pCMV-RBM39	F: CCAGTCGACTCTAGAGGATCCATGGCAGACGATATTGATATTGAAGC
	R: CAGGGATGCCACCCGGGATCCTCATCGTCTACTTGGAACCAGTAGTT
pCMV-c-Jun	F: CCAGTCGACTCTAGAGGATCCATGACTGCAAAGATGGAAACGAC
	R: CAGGGATGCCACCCGGGATCCTCAAAACGTTTGCAACTGCTGC
pET-28a-RBM39-X7	F: CAGCAAATGGGTCGCGGATCCATGGCAGACGATATTGATATTGAAGC
	R: GTGGTGGTGGTGGTGCTCGAGTCGTCTACTTGGAACCAGTAGTT
RRM1off	F: AAGAGATGCAGAAAAAAACAGAGCTGCAGC
	R: TGCATCTCTTTCCTCAGGAGTTAGATTATC
RRM2off	F: TGCTGGACCTCGTACTGATGCTTCCAGTGC
	R: AGGTCCAGCACTTCCCTTTTGTAGATTGTT
RRM3off	F: AACTGGTCGTCCAACTTATCACAACCTCTT
	R: ACGACCAGTTGTTCCCAAGTCAATTCCAGT
NLS-off	F: TGAAGCAATGCTAACTCCTGAGGAAAGAGA
	R: CATTGCTTCAATATCAATATCGTCTGCCAT

### Transient Transfection of Eukaryotic Expression Plasmid Into Cells

Previous to transient transfection, adherent HEK293T, HeLa or 3D4/21 cells were seeded in a 6/12/24-well plate (Corning Inc, Corning, NY, USA). Cells were transfected at the appropriate confluence with eukaryotic expression plasmid. Briefly, 10 µM polyethyleneimine (PEI) was preheated at 85°C and replace the old medium with Reduced-Serum Medium (Opti-MEM, Thermo Fisher Scientific) before transfection 1 h in advance. Add a suitable equal volume of medium to the two centrifuge tubes, then add the appropriate plasmid and PEI, shake and mix, and place at room temperature for 5 minutes. Then, mix and place them at room temperature for 20-30 minutes. Afterwards, add the mixture to the cells and incubate the cells in 37°C incubator containing 5% CO_2_. After 4-6 hours, discard the mixed solution and replace to complete medium, and continue to grow cells for 24-48 h.

### Western Blot

Cell samples were lysed by radioimmunoprecipitation assay (RIPA) (Solarbio) added with protease inhibitor cocktail phenylmethanesulfonyl fluoride (PMSF, Solarbio). Cells lysis supernatants were added to 5×loading buffer, following boiled for 10 min and separated by SDS-PAGE. The separated proteins were transferred onto a polyvinylidene fluoride (PVDF) blotting membrane (GE Healthcare) and blocked for 1h with 5% skimmed milk in 1×TBST (Tris-buffered saline containing 0.05% Tween-20) at room temperature, followed by incubation with the primary antibodies diluted in 1×TBST at 4°C overnight and secondary HRP-conjugated antibodies diluted in 1×TBST for 1h at room temperature. Finally, the membrane was detected through Pierce ECL Western Blotting Substrate (Thermo Scientific, Waltham, MA, USA) and exposed through Chemi Doc XRS Imaging System (BIO-RAD, USA).

### Quantitative Real-Time PCR

Quantification of the relative levels of gene expression was performed using qRT-PCR. Total RNAs were extracted by RNAiso plus (Takara). The cDNA was synthesized with EasyScript First-Strand cDNA Synthesis SuperMix according to the manufacturer’s instructions (TransGene). The relative levels of gene expression were analyzed by QTOWER3G IVD Fluorescent Quantitative PCR System (Jena, Germany) using TransStart Top Green qPCR SuperMix (TransGene) with three-step amplification and the data were calculated by the comparative cycle threshold (C_T_) method (2^−ΔΔCt^). The thermal cycler program consisted of 95°C for 10 min and then 45 cycles at 95°C for 15 s, 58°C for 30 s, and 72°C for 30 s. The β-actin was used as an internal control for normalization. All the primers used for quantitative real-time PCR are shown in **Table 2**.

**Table 2:** Primers used in quantitative real-time PCR.

Primer name	Primer sequence (5’–3’)
Q-RBM39	F: CCAAATGCCAAAGAACC
	R: GAATGTGCCAAGAAAGC
Q-nsp2	F: CAGCCTTATGACCCCAACCAG
	R: TGGGCAAAGTCCCCTGTACCAA
Q-N	F: CAGTCAATCAGCTGTGCCAAA
	R: ATCTGACAGGGCACAAGTTCCA
Q-C-Jun	F: ACGACCTTCTACGACGATG
	R: TGGTGATGTGCCCGTTA
Q-β-actin	F: CAAATGCTTCTAGGCGGACT
	R: TGCTGTCACCTTCACCGTTC
Q-IFN-β	F: GCAGTATTGATTATCCACGAGA
	R: TCTGCCCATCAAGTTCCAC
Q-NF-κB	F: GTGTGTAAAGAAGCGGGACCT
	R: CACTGTCACCTGGAAGCAGAG
Q-TNF-α	F: GAGATCAACCTGCCCGACT
	R: CTTTCTAAACCAGAAGGACGTG
Q-IL-6	F: ACTGGCAGAAAACAACCTGA
	R: CCTCGACATTTCCCTTATTGCT
Q-IL-1β	F: TGTTCTGCATGAGCTTTGTG
	R: TCTGGGTATGGCTTTCCTTAG

### Dual Luciferase Reporter Assay

HEK293 T cells plated to 24-well plates at 70%-80% confluence were transfected with RBM39, AP-1 luciferase reporter plasmid, PRRSV-JXwn06 infectious clone plasmid, siRBM39 or sic-Jun (Sigma) and the Renilla luciferase control reporter plasmid was used as a reference. The cells were collected and lysed at 24 h post-transfection, and the cell supernatant was used to detect luciferase activity through Dual Luciferase Reporter Gene Assay Kit (Yeasen Technology) according to the manufacturer’s instructions.

### Immunoprecipitation (IP) and Co-Immunoprecipitation (co-IP) Assays

HEK293 T Cells cultivated in six-well plates were transfected or co-transfected with appropriate eukaryotic expression plasmid. The cells were harvested and resuspended by PBS 24 h post-transfection, and lysed on ice for 15 min in 400 µl RIPA lysis buffer. Then the samples were centrifuged at 12,000 rpm for 5 min. We took out the 50 μL supernatant in new tube, added 10 μL loading buffer and boiled them to make input samples. Subsequently, anti-Flag-labeled beads (Sigma) were activated by RIPA and added the rest of the cell lysis supernatant. The reaction mixture was incubated at 4°C overnight. The beads were washed three times with lysis buffer containing PMSF for 5 min each time and added 50 μL lysates and 10 μL loading buffer, boiled for 10 min to make IP samples. Finally, the proteins bound to the beads were separated *via* SDS-PAGE, transferred to PVDF membrane, and detected with the proper antibodies.

### Indirect Immunofluorescent Assay (IFA) and Confocal Microscopy

Adherent HEK293T, HeLa or 3D4/21 cells grown on glass coverslips in 12-well plates at 30% to 50% confluence were transfected with plasmids expressing RBM39 and/or c-Jun. The empty vector plasmid was used as a negative control (NC). Subsequently, 3D4/21 cells were infected with PRRSV at a multiplicity of infection (MOI) of 0.4. At 24 h post-transfection or post-infection, the cells washed with 1×phosphate-buffered saline (PBS), fixed with 4% paraformaldehyde for 30 min at room temperature, permeabilized with 0.5% Triton X-100 for 15 min and blocked with 5% bovine serum albumin (BSA) dissolved in 1×PBS containing 0.05% of Tween-20 (PBST) for 2h at room temperature. Anti-HA (hemagglutinin), anti-Flag and anti-PRRSV-N primary antibodies were diluted in 2% BSA and incubated with slides overnight at 4°C. Following a wash performed with PBS 3 times for 5 min each time, the cells were incubated with fluorescein isothiocyanate (FITC)-conjugated goat anti-mouse/rabbit IgG or IF555-conjugated goat anti-mouse IgG secondary antibody diluted in 2% BSA at 37°C for 1 h in a humidified chamber. The slides were washed with PBST and nuclei were counterstained with Hoechst 33258 dye (Solarbio) for 5 min. Confocal images were collected with confocal laser scanning microscope (UltraView Vox, PerkingElmer) and taken at 40 × or 100 × magnification (Olympus).

### RNA Interference

A small interfering RNA (siRNA) assay, which was synthesized from Sigma (**Table 3**), was performed through siRNA targeting the knockdown of RBM39 (siRBM39) or c-Jun (sic-Jun) gene, and negative control (NC) was used as control groups. For siRNA transfection, 3D4/21 cells plated in 12-well plates and grown to 70%-80% confluence were transfected with 50 nmol of siRNAs using 10 µM PEI. The cells were infected with PRRSV at 0.4 MOI 24 h post-transfection and incubated for additional 24 h. Subsequently, cells were collected for qRT-PCR and Western blotting analysis to detect the expression levels.

**Table 3:** Primers used in RNA interference assay.

Primer name	Primer sequence (5’–3’)
Negative control (RBM39)	F: UUCUCCGAACGUGUCACGUTT
	R: ACGUGACACGUUCGGAGAATT
siRNA-RBM39-1308	F: UUCCUGCGAGCUCAAAUCCTT
	R: GGAUUUGAGCUCGCAGGAATT
siRNA-RBM39-1631	F: AAACAUGUUAGAGAGUUGGTT
	R: CCAACUCUCUAACAUGUUUTT
Negative control (c-Jun)	F: UUCUCCGAACGUGUCACGUTT
	R: ACGUGACACGUUCGGAGAATT
siRNA-c-Jun-197	F: AGGUCGUUUCCAUCUUUGCTT
	R: GCAAAGAUGGAAACGACCUTT
siRNA-c-Jun-261	F: UUACUGUAGCCGUAGGCACTT
	R: GUGCCUACGGCUACAGUAATT

### RNA Immunoprecipitation (RIP)

3D4/21 cells plated in 6-well plates and grown to 70%-80% confluence were transfected with 2μg HA-RBM39 or Flag-RBM39 plasmids. Then the cells were infected with 0.4 MOI PRRSV 12 h after transfection. After another 24 hours, the cells were collected and lysed with 400 μl RIPA supplemented with murine RNase inhibitor (1:100). Part of the supernatant was used for immunoblotting analysis, and the remaining supernatant was incubated with HA/Flag-tags beads overnight at 4°C. The co-precipitated RNAs were extracted, reverse transcribed and detected through PCR *via* corresponding primers.

### Statistical Analysis

Data were presented as the mean of three independent replicates. Statistical significance was analyzed by the unpaired t-test using Origin and GraphPad Prism software. Statistical differences were considered to be statistically significant at P-value < 0.05 (*p < 0.05; **p < 0.01; ***p < 0.001; ****p<0.0001).

## Results

### Upregulation of RBM39 in 3D4/21 Cells by PRRSV Infection

**Figure 1:**
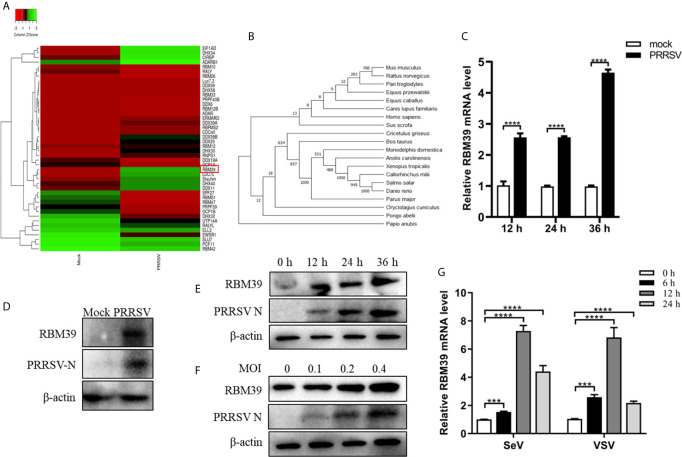
Upregulation of RBM39 in 3D4/21 cells by PRRSV infection. **(A)** Heat map of RNA binding protein differentially expressed genes made using online websites (http://www.heatmapper.ca/). **(B)** RBM39 evolutionary tree among different species. **(C–E)** 3D4/21 cells were subjected to mock infection or infected with 0.4 multiplicity of infection (MOI) PRRSV. **(C)** Cells were collected at the three time points (12, 24, 36 h) and subjected to real-time RT-PCR to analyze the expression level of RBM39. **(D)** Cell lysates were collected at 24 h post-infection and subjected to Western blot analysis with antibody to RBM39 to analyze the protein expression. **(E)** The cells were collected at 0 h, 12 h, 24 h and 36 h post-infection; **(F)** The cells were infected with 0, 0.1, 0.2 and 0.4 MOI PRRSV respectively and were collected at 24 h post-infection, the RBM39 protein level was analyzed by WB. **(G)** 3D4/21 cells were infected with SeV and VSV. The cells were collected at 0, 6, 12, and 24 h post-infection, and the RBM39 mRNA level was analyzed by qRT-PCR. Data are representative of results from three independent experiments. The statistically significant at P-value was demonstrated in *Statistical Analysis*.

PRRSV infection can result to upregulation or downregulation of many genes in cells. To find out genes that displayed significant changes in transcription after PRRSV infection, a transcriptome sequencing analysis derived from PAM cell was performed. RBM39 was found to be significantly upregulated after PRRSV infection by statistical analysis (**Figure 1A**). Through the analysis of RBM39 gene domain, it is known that the protein is conserved in mammals (**Figure 1B**). To investigate the levels of RBM39 accumulation during viral infection, we infected 3D4/21 and PAM cells with PRRSV at 0.4 MOI. After collecting cells and extracting total RNA at different time points (12 h, 24 h and 36 h) post-infection, mRNA abundance of RBM39 was analyzed by quantitative reverse transcription-PCR (qRT-PCR). Compared with the control group, the mRNA levels of RBM39 significantly increased at three different time points after PRRSV infection (**Figure 1C**). Furthermore, to analyze the effect of PRRSV infection on the protein expression level of RBM39 gene, we collected cells infected with PRRSV for different time periods (0 h, 12 h, 24 h and 36 h; 0.4 MOI) or at different multiplicity of infection (0, 0.1, 0.2 and 0.4; 24 h), followed by Western blot analysis with antibody to RBM39. The results showed that the protein expression in the cellular level of RBM39 after PRRSV infection was a dramatic increase compared with control groups (**Figures 1D** and **S2A**) and increased over time (**Figures 1E** and **S2B**). In addition, the results displayed that the protein expression level of RBM39 increased in a dose-dependent manner on MOI (**Figures 1F** and **S2C**). To verify the expression level of RBM39 after infection with other RNA viruses, we infected cells with SeV and VSV, and then extracted the total RNA of the cells at 0, 6, 12 and 24 h after infection, and analyzed the mRNA level of RBM39 by qRT-PCR. The results showed that RBM39 mRNA levels increased significantly at four time points (**Figure 1G**).

### RBM39 Contributes to PRRSV Proliferation

**Figure 2:**
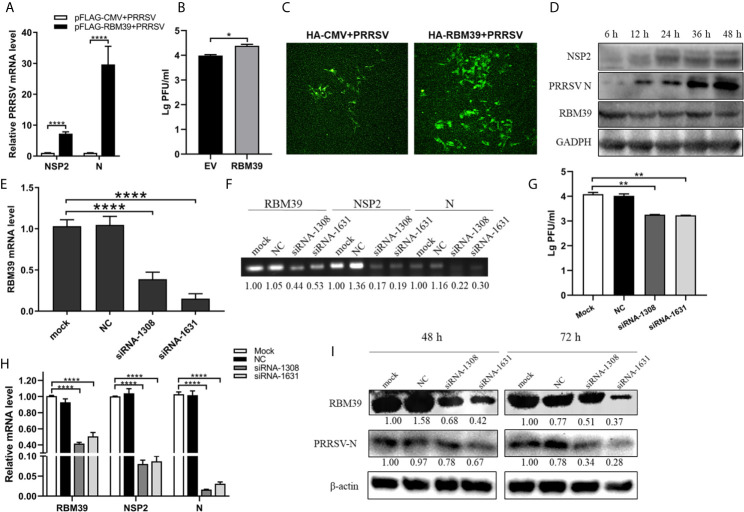
RBM39 contributes to PRRSV proliferation. **(A–D)** 3D4/21 cells were transfected with Flag/HA-RBM39 or empty vector and infected with PRRSV at 12 h post-transfection. The infected cells were collected at the indicated times post-infection. **(A)** The mRNA loads of PRRSV N and nsp2 were tested by qRT-PCR. Detection of viral titers of PRRSV in cell supernatants of RBM39 overexpressed **(B)** or interfered **(G)** samples. **(C)** The cell slide was incubated overnight at 4°C with the antibody of PRRSV N and then incubated with FITC –labeled secondary antibody conjugate. The expression of PRRSV N was detected by Laser confocal fluorescence microscope. **(D)** PRRSV nsp2 and N protein expression were tested by WB. **(E–I)** The results showed 3D4/21 cells transfected with siRNA of RBM39, negative control (NC) or untransfected mock cells. The cells were infected with PRRSV at 12 h post-transfection and collected at 24 h post-infection. The mRNA levels of RBM39 **(E, H)**, PRRSV N and nsp2 **(H)** were detected by qRT-PCR. **(F)** The agarose gel electrophoresis analysis after qRT-PCR. **(I)** RBM39 and PRRSV protein expression levels were examined by Western blot after 48 or 72 h. Data are representative of results from three independent experiments. The statistically significant at P-value was demonstrated in *Statistical Analysis*.

The effect of RBM39 on the virus has not been reported before. To explore the effect of RBM39 on PRRSV proliferation, the 3D4/21 and PAM cells were transfected with Flag-RBM39 expression plasmids or empty vector, followed by infected with 0.4 MOI PRRSV 12 h post-transfection. Total RNA was extracted from the cells at 24 h post-transfection, and subsequently, transcription levels of PRRSV nsp2 and N gene were analyzed by qRT-PCR. Compared with the control groups, the overexpression of RBM39 promoted the mRNA level of the PRRSV nsp2 and N gene (**Figures 2A** and **S2D**). Meanwhile, the virus titer in cells after transfection of the RBM39 plasmid was higher than the titer seen with the control cells (**Figures 2B** and **S2E**). Confocal microscopy showed that the RBM39 overexpression group can observe more green fluorescent signals in the field of view than the empty vector group (**Figure 2C**). To further figure out the enhancement of RBM39 on PRRSV, we collected cells at five different time point (6 h, 12 h, 24 h, 36 h, 48 h) after PRRSV infection respectively, and subjected to Western blot analysis with antibody to N and nsp2. The results of Western blot analysis revealed that the protein expression level of N and nsp2 increased with time (**Figure 2D**). Subsequently, to further verify the effect of RBM39 on viral proliferation, we designed RBM39 small interfering RNA (siRNA) that targeted RBM39 gene and inhibited the transcription and protein expression of RBM39. Consistent with previous results, knockdown of RBM39 reduced the PRRSV proliferation to some extent at the virus titer, transcription and protein levels (**Figures 2E–I**). The results indicate that changes in the transcription and translation levels of RBM39 can affect PRRSV proliferation and that RBM39 contributes to PRRSV proliferation.

**Figure 3:**
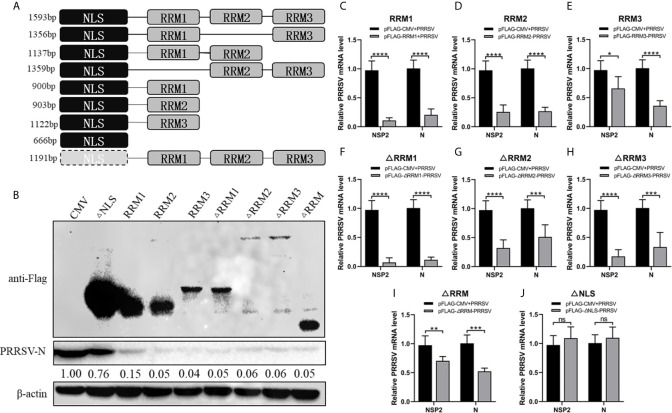
The RRM domain of RBM39 is important to promote the proliferation of PRRSV. **(A)** The constructed 8 truncated forms of RBM39 *via* the reverse amplification method, namely Flag-RRM1, RRM2, RRM3, ΔRRM1, ΔRRM2, ΔRRM3, ΔRRM, ΔNLS. **(B–J)** 3D4/21 cells were transfected with 8 truncations and infected with PRRSV after 12 h. After 24 hours, the proliferation of PRRSV was detected by Western Blot analysis **(B)** and qRT-PCR **(C–J)**. Data are representative of results from three independent experiments. The statistically significant at P-value was demonstrated in *Statistical Analysis*. ns, no significant difference.

In order to determine the effect of the three RRM domains of RBM39 on the proliferation of PRRSV, we constructed eight truncated forms of RBM39 *via* the reverse amplification method, namely Flag-RRM1, RRM2, RRM3, ΔRRM1, ΔRRM2, ΔRRM3, ΔRRM, ΔNLS (**Figure 3A**). It is worth noting that the DNA sequence that connects each domain is preserved. 3D4/21 cells were transfected with these eight truncations and infected with the virus. After 24 hours, the proliferation of PRRSV was detected by qPCR and Western blot analysis. The results of qPCR and Western Blot analysis showed that compared with the control group, the proliferation of PRRSV in the experimental group transfected with Flag-RRM1, RRM2, RRM3, ΔRRM1, ΔRRM2, ΔRRM3, ΔRRM plasmid was inhibited (**Figures 3B–J**). However, the proliferation of PRRSV in the group transfected with Flag-ΔNLS had no significant difference (**Figures 3B, J**). It shows that the three RRM domains of RBM39 play an important role in promoting the proliferation of PRRSV.

### The Promotion of PRRSV Proliferation by RBM39 Is Related to Innate Immunity

**Figure 4:**
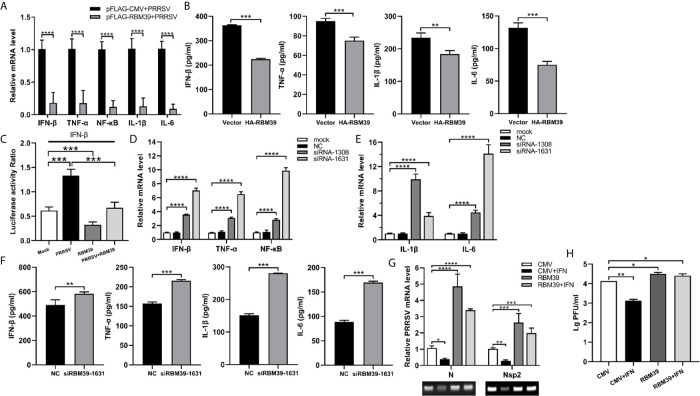
The promotion of PRRSV proliferation by RBM39 is related to innate immunity. 3D4/21 cells were transfected with Flag-RBM39 or empty vector plasmid **(A, B, G, H)**, siRNA of RBM39 or NC **(D–F)** and the infected with PRRSV 12 h post-transfection. The mRNA levels of IFN-β, TNF-α, NF-κB, IL-1β, IL-6 were determined by qRT-PCR at 24 h post-infection **(A, D, E)**. Cytokine detection in the condition of RBM39 overexpressed **(B)** or interfered **(F)** by ELISA kits (Solarbio). **(C)** HEK293 T cells were transfected or co-transfected with HA-RBM39 and PRRSV infectious clone plasmid. The luciferase activity of IFN-β promoter reporter gene was measured through Dual Luciferase Reporter Gene Assay Kit (Yeasen Technology). **(G)** The cells were treated with IFN post-infection. After another 24 h, mRNA levels of PRRSV N and nsp2 were tested by qRT-PCR. **(H)** Detection of viral titers of PRRSV in cell supernatants after IFN treated. Data are representative of results from three independent experiments. The statistically significant at P-value was demonstrated in *Statistical Analysis*.

Previous studies showed that RBM39 is beneficial to PRRSV proliferation. To test the possibility of RBM39 negative regulatory effect on innate immunity, we examined the mRNA levels and concentration of some transcription factors and cytokines related to innate immunity, such as NF-κB, TNF-α, IFN-β, IL-1β, IL-6. Cytokine concentration is detected by ELISA kit (Porcine TNF-α/IFN-β/IL-1β/IL-6 ELISA KIT, Solarbio). The results showed that overexpression of RBM39 drastically decreased the transcription and secretion levels of these genes in 3D4/21 cells (**Figures 4A, B**). To investigate whether RBM39 impacts IFN-β-related signaling pathway, HEK293T cells were transfected with HA-RBM39 and PRRSV infectious clone plasmid or co-transfected with them. Subsequently, we measured the activity of the IFN-β promoter reporter gene and found that the activation was increased by PRRSV stimuli but reduced by RBM39 overexpression compared with the control group (**Figure 4C**). Most important of all, when RBM39 and PRRSV co-transfected, the activity is lower than that of the group transfected with PRRSV alone, indicating that the overexpression of RBM39 may promote PRRSV proliferation by inhibiting the IFN-β signaling pathway (**Figure 4C**). In addition, the mRNA and secretion levels of these genes related to innate immunity were upregulated when expression of RBM39 was disturbed by the introduction of siRNA (**Figures 4D–F**). Ulteriorly, we found out that RBM39 was capable of attenuating the inhibitory effect of interferon on PRRSV (**Figure 4G**) and increasing the virus titer at the same time (**Figure 4H**). In conclusion, these data demonstrated that RBM39 might negatively regulate antiviral immune responses and resulting in the promotion of PRRSV proliferation.

### Interaction and Co-Localization Between RBM39 and c-Jun

**Figure 5:**
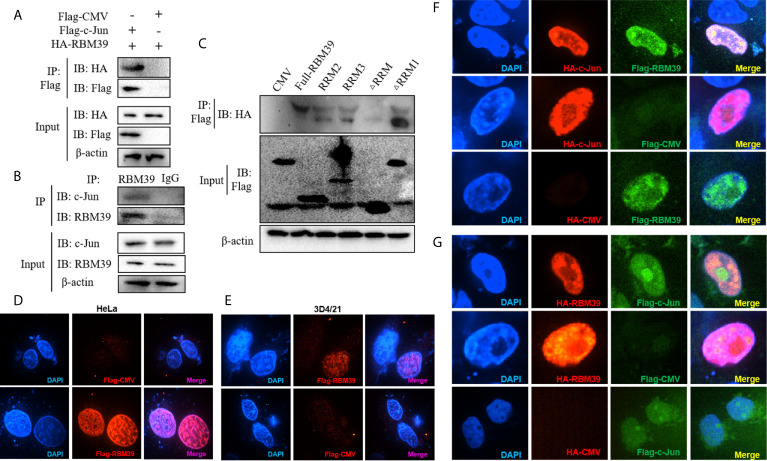
Interaction and co-localization between RBM39 and c-Jun. Interaction between RBM39 and c-Jun in the condition of overexpressed **(A)** or endogenous expression **(B)**. **(C)** HEK293 cells were first transfected with HA-c-Jun and then co-transfected with Flag-RBM39 (full-length), RRM2, RRM3, ΔRRM1, ΔRRM plasmid or empty vector respectively. The cell lysates were then immunoprecipitated with an anti-HA MAb and detected by Western blot at 24h post-transfection. **(D–G)** HEK293T cell were co-transfected with plasmid Flag-RBM39+HA-c-Jun or Flag-c-Jun+HA-RBM39 plasmid. After 24 h, cells were fixed by 4% paraformaldehyde and doubly stained with rabbit anti-HA mAb and mouse anti-Flag antibody followed by FITC-conjugated goat anti-rabbit IgG (green) or IF555-conjugated goat anti-mouse IgG (red) antibody. Nuclei were stained with Hoechst 33258 dye (blue). Interaction and nuclear localization of RBM39 and c-Jun were observed using Laser confocal fluorescence microscope. Data are representative of results from three independent experiments.

Previous research and mass spectrometry (MS) results found that RBM39 binds and interact with the AP-1 component c-Jun (**Figures 5A, B**) (14). In addition, the data displays that the cryptic autonomous transactivation domain (AD) of RBM39 combined and interacted with the basic region-leucine zipper (bZIP) of c-Jun (14). In previous studies, the interaction region of RBM39 included the end of the RRM2 domain, the whole RRM3 domain, and the sequence connecting the two domains in the middle. Therefore, we are curious about whether RBM39 was capable of interacting with c-Jun when there is only RRM2 or RRM3. Afterwards, the plasmids HA-c-Jun and Flag-RBM39 (full-length), RRM2, RRM3, ΔRRM1, ΔRRM or empty vector was co-transfected into HEK293T respectively. Subsequently, cells were harvested after 24 h and interaction between RBM39 and c-Jun was detected *via* co-immunoprecipitation. The results showed that c-Jun can interact with full-length RBM39, RRM2, RRM3 and ΔRRM1 but not ΔRRM and empty vector (**Figure 5C**). It is worth noting when both RRM2 and RRM3 domains exist, the interaction between RBM39 and c-Jun is stronger than only one of them exists (**Figure 5B**). To observe the co-localization of the two proteins in the cell, we co-transfected plasmids Flag-RBM39 + HA-c-Jun or Flag-c-Jun + HA-RBM39 into HEK293T cell lines. Confocal microscopy showed that both RBM39 and c-Jun expressed and co-localized in the nucleus, while similar results were not observed in the control groups (**Figures 5D–G**).

### RBM39 Down-Regulates AP-1 Signaling Pathway

**Figure 6:**
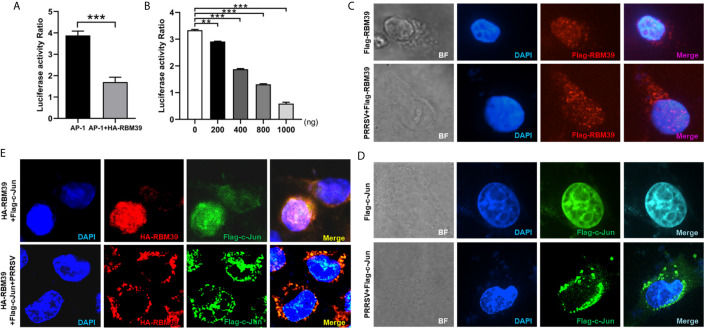
RBM39 down-regulates AP-1 signaling pathway and PRRSV infection triggers out of the nucleus of translocation of RBM39 and c-Jun. **(A, B)** HEK293 T cells were transfected or co-transfected with AP-1 promoter reporter plasmid and different concentrations of HA-RBM39. The luciferase activity of AP-1 promoter reporter gene was measured through Dual Luciferase Reporter Gene Assay Kit (Yeasen Technology). **(C–E)** The 3D4/21cells were respectively transfected or co-transfected with HA-RBM39 and Flag-c-Jun plasmids, the cells were infected with 0.4 MOI PRRSV 12 h post-transfection. After 24 h, cells were incubated with rabbit anti-HA mAb and mouse anti-Flag antibody followed by FITC-conjugated goat anti-rabbit IgG (green) or IF555-conjugated goat anti-mouse IgG (red) antibody. Nuclei were stained with Hoechst 33258 dye (blue). Interaction and nuclear localization of RBM39 and c-Jun were observed using Laser confocal fluorescence microscope. Data are representative of results from three independent experiments. The statistically significant at P-value was demonstrated in *Statistical Analysis*.

The c-Jun is an important part of AP-1 signaling pathway and a significant immune-related protein. To investigate whether RBM39 impacts AP-1 signaling pathway, HEK293 T cells were transfected or co-transfected with HA-RBM39 and AP-1 promoter reporter plasmid. The results display that the activity of AP-1 promoter reporter gene was reduced by RBM39 overexpression in a dose-dependent manner (**Figures 6A, B**).

To investigate whether PRRSV infection can cause the intracellular localization changes of RBM39 and c-Jun in 3D4/21 cells, the cells were respectively transfected with HA-RBM39 and Flag-c-Jun plasmids, 12 h after transfection, cells were infected with 0.4 MOI PRRSV. In the subsequent immunofluorescence analysis, we noticed that RBM39 or c-Jun proteins were localized in the nucleus ahead of PRRSV infection (**Figures 6C–E**, up). After the virus infection, we observed that both RBM39 and c-Jun proteins had nuclear-cytoplasmic trafficking and co-localization (**Figures 6C–E**, down). These results illustrated that PRRSV infection caused the nuclear export of RBM39 and c-Jun and this phenomenon may be related to the mechanism of RBM39 promoting PRRSV proliferation.

### RBM39 Impair Phosphorylation of c-Jun to Down-Regulation of AP-1 Signaling Pathway

**Figure 7:**
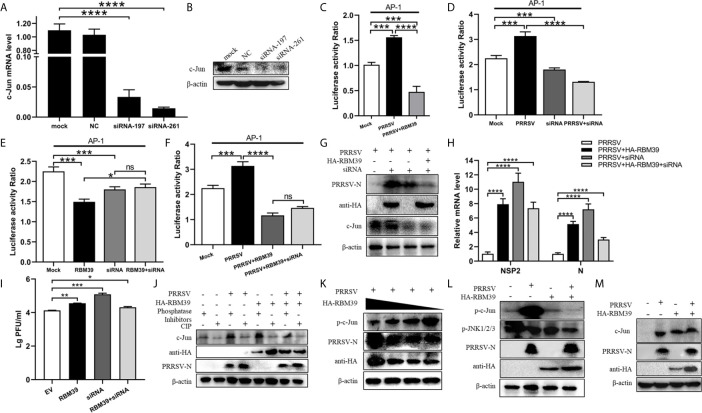
RBM39 affect phosphorylation of c-Jun to down-regulation of AP-1 signaling pathway. 3D4/21 cells were transfected with siRNA of c-Jun or the NC, and the mRNA and protein levels of c-Jun were examined by qRT-PCR **(A)** and WB analysis **(B). (C–F)** HEK 293T cells were transfected or co-transfected with RBM39, siRNA of c-Jun, PRRSV infectious clone plasmid, AP-1/Renilla luciferase reporter plasmids. At 24 h after transfection, the luciferase activity of AP-1 promoter reporter gene was measured through Dual Luciferase Reporter Gene Assay Kit (Yeasen Technology). **(H, J)** 3D4/21 cells were transfected or co-transfected with RBM39 and siRNA of c-Jun, respectively. The cells were infected with o.4 MOI PRRSV 12 h post-transfection and collected another 24 h post-infection. The protein levels of PRRSV N were detected by WB **(G)** and the mRNA levels of PRRSV N and nsp2 were tested by qRT-PCR **(H)**. **(I)** Detection of viral titers of PRRSV in cell supernatants of **(H)** each experiment group samples. **(J)** The c-Jun protein level was detected in the condition of phosphatase Inhibitors/CIP treated by WB analysis. **(K)** 3D4/21 cells were transfected with different concentrations of RBM39 (2µg, 1µg, 1.5µg, 0.5µg). The cell lysates were incubated with Rabbit anti-phospho-c-Jun (Ser73) mAb and detected by Western blotting. **(L, M)** 3D4/21 cells were transfected or untransfected with RBM39. 12 h after transfection, cells were infected or no infected with PRRSV. The protein levels of p-c-Jun, p-JNK1/2/3 **(L)** and c-Jun **(M)** were analyzed by immunoblotting. Data are representative of results from three independent experiments. The statistically significant at P-value was demonstrated in *Statistical Analysis*. ns, no significant difference.

To test whether RBM39 down-regulates the AP-1 pathway by affecting c-Jun, we designed siRNA to knock down the expression of c-Jun (**Figures 7A, B**). First, PRRSV infection triggers the activation of the AP-1 signaling pathway (**Figures 7C, D, F**), and RBM39 overexpression (**Figure 7E**) and c-Jun knockdown (**Figures 7C, F**) led to a decrease in the activity of the AP-1 promoter reporter gene (**Figures 7D, E**). On this basis, we hypothesized that the down-regulation of AP-1 activity is not relevant to the interaction between RBM39 with c-Jun; therefore, RBM39 and siRNA-c-Jun could reduce AP-1 activity to a greater degree than either. However, the results showed that RBM39 and siRNA-c-Jun did not have a synergistic effect with each other (**Figure 7E**), which indirectly indicates that the down-regulation of the AP-1 pathway by RBM39 is achieved by affecting c-Jun. Similarly, the activity of AP-1 promoter reporter gene was increased by PRRSV stimuli but reduced by RBM39 and PRRSV co-transfection compared with the control group (**Figures 7C, D, F**). However, compared with the co-transfection of RBM39 and PRRSV, the activity of AP-1 was not significantly reduced in the additional condition of c-Jun knockdown (**Figure 7F**), indicating that knockdown of c-Jun has not a synergistic effect on the AP-1 activity reduction caused by RBM39 (**Figure 7F**), further indicating the down-regulation of the AP-1 pathway by RBM39 is related to c-Jun.

To further prove the above conclusion, we tested the effect of c-Jun knockdown and RBM39 on PRRSV proliferation. The results illustrate that it is obvious that RBM39 and siRNA-c-Jun alone can promote the proliferation of PRRSV (**Figures 7G–I**). However, when the RBM39 and siRNA-c-Jun were co-transfected, the promotion of PRRSV proliferation not only did not increase synergistically, but decreased, indicating that the promotion of PRRSV proliferation by RBM39 is through interacting with c-Jun (**Figures 7G–I**).

In order to detect the effect of RBM39 on the phosphorylation level of proteins in the cell signaling pathway caused by PRRSV infection, 3D4/21 cells were transfected or co-transfected with PRRSV and HA-RBM39 plasmids, phosphatase Inhibitors/CIP was used to treat cell lysate supernatant and c-Jun antibody was used to detection for Western Blot analysis. In addition, phosphorylated antibody was used to detect the phosphorylation level of the protein 24 hours post-infection. The results showed that c-Jun was heavily phosphorylated after PRRSV (**Figure 7J**), which triggered subsequent immune responses. However, under the condition of RBM39 overexpression, the phosphorylation of c-Jun caused by PRRSV infection was greatly reduced and in a dose-dependent manner (**Figures 7K, L**). What is noteworthy is that the c-Jun and phosphorylation level of JNK did not decrease significantly in the co-transfection group (**Figures 7L, M**), indicating that RBM39 may not affect the phosphorylation of JNK and its upstream molecules triggered by PRRSV infection. In summary, the overexpression of RBM39 reduces the phosphorylation level of c-Jun after PRRSV infection, and thus down-regulates the AP-1 signaling pathway. This may be the mechanism by which RBM39 promotes PRRSV proliferation.RBM39 Binds to PRRSV RNA

**Figure 8:**
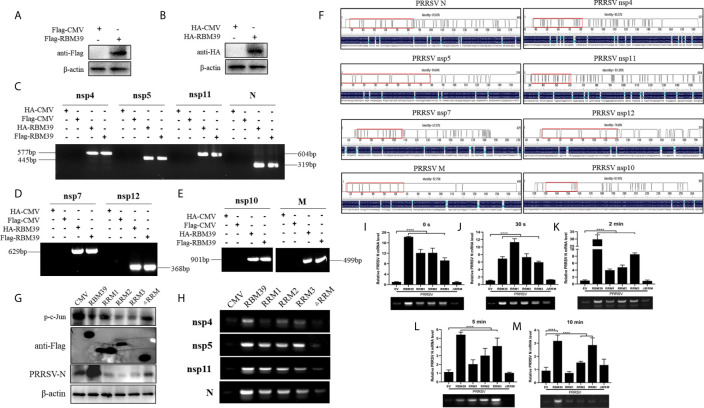
RBM39 binds to PRRSV RNA. **(A, B)** 3D4/21 cells were transfected with Flag/HA-RBM39 and infected with PRRSV 12 h after transfection. After another 24 hours, the cells were detected for immunoblotting analysis with anti HA/Flag mAb. **(C–E)** The agarose gel electrophoresis of PRRSV nsp4, nsp5, nsp7, nsp10-12, M and N PCR products after extracted RNA was reverse transcribed and amplified with corresponding primers. **(F)** Comparison of sequencing results using DNAMAN. **(G–M)** 3D4/21 cells were transfected with Flag-RBM39, RRM1, RRM2, RRM3, ΔRRM, or empty vector respectively and infected with PRRSV 12 h after transfection. After another 24 hours, the cells were tested for immunoblotting **(G)** and RIP analysis. **(I–M)** The PRRSV N level was analyzed by qRT-PCR after RIP. **(H)** The agarose gel electrophoresis of PRRSV nsp4, nsp5, nsp11, and N PCR products. Data are representative of results from three independent experiments. The statistically significant at P-value was demonstrated in *Statistical Analysis*.

RBM24, which belongs to the same RNA-binding protein family as RBM39, has RNA-binding function and can bind to the 5’- and 3’-terminal ends of HBV RNA (37). Here, the RBM39 binds to viral RNA were also identified. To verify whether RBM39 can bind to PRRSV RNA, 3D4/21 cells were transfected with HA-RBM39 or Flag-RBM39 plasmids and infected with PRRSV 12 h after transfection. After another 24 hours, the cells were collected and lysed with RIPA supplemented with murine RNase inhibitor (40U/µl, Yeasen). Part of the supernatant was used for immunoblotting analysis (**Figures 8A, B**), and the remaining supernatant was carefully added to HA/Flag-tags beads and combined overnight at 4°C. After RNA was extracted and reverse transcribed, it was amplified with corresponding primers, and a single band with the same size as predicted was recovered and sequenced (**Figures 8C–E**). Through the alignment between sequenced sequence and known sequence, we found that we correctly amplified nsp4 (85.27%), nsp5 (87.87%), nsp7 (67.57%), nsp10 (81.91%), nsp11 (91.39%), nsp12 (74.60%), M (92.79%), and N (94.04%) genes of PRRSV (**Figure 8F**), and indicating that RBM39 could bind to PRRSV RNA. To further explore the effects of the three RRM domains on binding to PRRSV RNA and c-Jun phosphorylation, we transfected RBM39, RRM1, RRM2, RRM3, ΔRRM plasmids, or empty vector into 3D4/21 cells. 12 h after transfection, cells were infected with PRRSV. After another 24 hours, the cells were collected and subjected to RIP and immunoblotting. The results show that the deletion of RRM2 or RRM3 has a significant inhibitory effect on c-Jun phosphorylation (**Figure 8G**), indicating that RRM2 and RRM3 may be important domains to reduce c-Jun phosphorylation. To verify whether RBM39 has a protective effect on PRRSV RNA, we heated each experimental group at 85°C for different time periods (0 s, 30 s, 2 min, 5 min, 10 min) after RIP, and then level of PRRSV N was detected by qPCR. Presence of the full length of RBM39 or only one RRM domain, it has a certain degree of protection against PRRSV RNA. However, there is no significant difference in absence of three RRM domains (**Figures 8I–M**). It is worth noting that with the extension of time, the protective ability of PRRSV RNA tends to weaken overall. Moreover, we tried to amplify the RNA extracted after each group of RIP, and the results showed that nsp4, nsp5, nsp11, and N were obtained (**Figure 8H**), indicating that three RRM domains of RBM39 are crucial to RNA binding capacity to further impact viral proliferation.

## Discussion

RBM39 is an RNA-binding protein that has the function of binding RNA. In this study, we learned that RBM39 binds to PRRSV RNA and can promote its proliferation in 3D4/21 cells. According to reports, as the same RNA binding protein, RBM24 binds to the 3’-TR of HBV and stabilizes the stability of viral RNA (37). Therefore, we speculate that the upregulated cytoplasmic RBM39 promotes the combination of RBM39 with PRRSV RNA and further improve the viral RNA stability. In addition, the function of RBM39 in pre-mRNA splicing and its effects on the promotion of PRRSV proliferation should be further considered.

**Figure 9:**
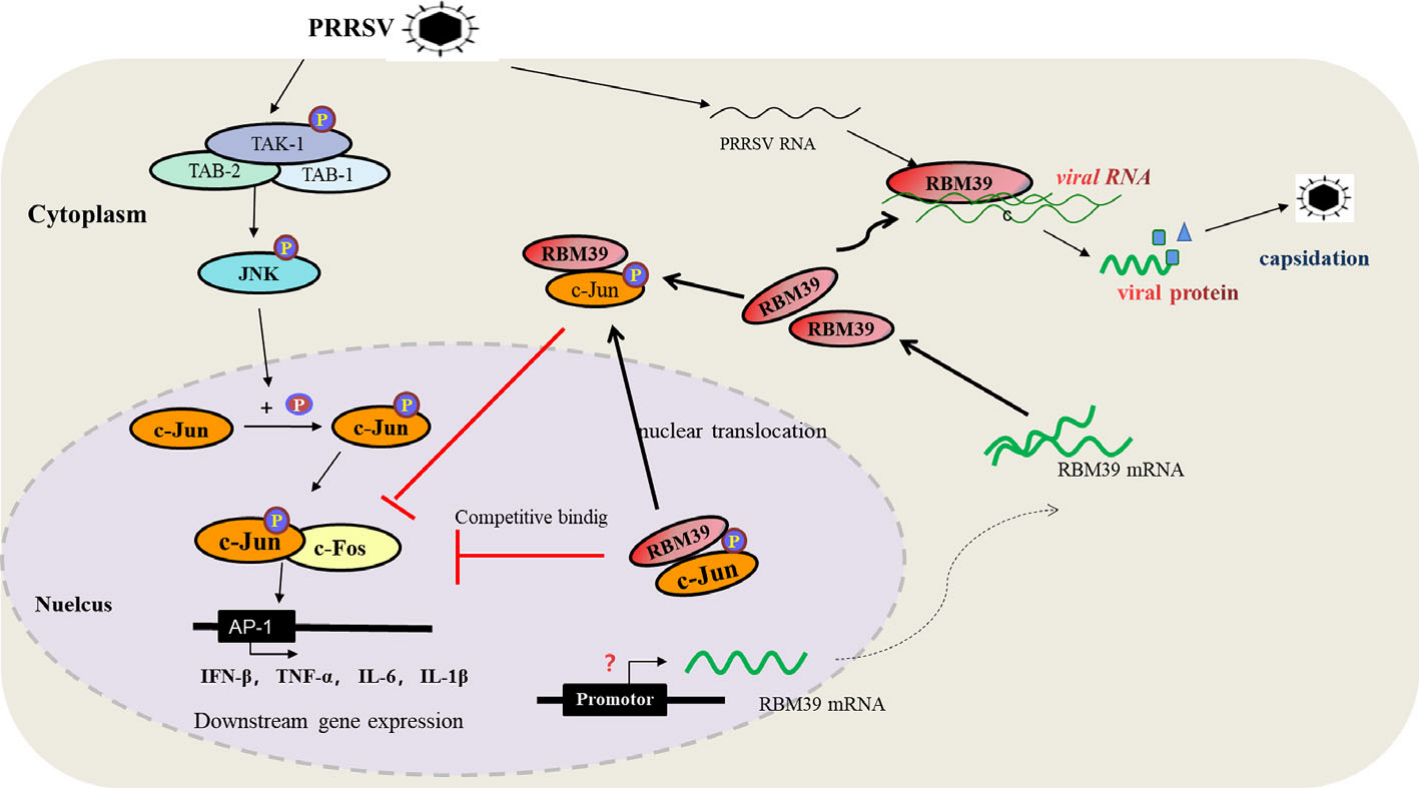
The diagram of the mechanism of RBM39 promoting PRRSV proliferation. After PRRSV infection, the combination of RBM39 and c-Jun translocates to the outside of the nucleus together under certain effects, which hinders the phosphorylation of c-Jun by JNK and reduces the level of c-Jun phosphorylation. At the same time, RBM39 competes with c-Fos to bind c-Jun and robs c-Jun of AP-1 dimer, which reduces the number of AP-1 dimers, and then down-regulates the activity of AP-1 pathway and its downstream signals, and finally, promotes the proliferation of PRRSV. In addition, the combination of RBM39 and PRRSV RNA may promote PRRSV proliferation to some extent.

In summary, we report that porcine RBM39 negatively regulate the AP-1 signaling pathway and promote PRRSV proliferation. The mechanism by which RBM39 promotes PRRSV proliferation can be summarized as follows (1). After PRRSV infection, RBM39 is up-regulated, which promotes the formation of RBM39 and c-Jun complex in the cytoplasm and cytoplasmic retention. (2) PRRSV infection makes JNK phosphorylation incorporated into the nucleus, and promotes the phosphorylation of nuclear transcription factor c-Jun; RBM39 and the phosphorylated c-Jun in the nucleus form a complex that is prone to translocation from the nucleus to the cytoplasm, which further reduces the retention of c-Jun in the nucleus; Decreased phosphorylated c-Jun in the nucleus, down-regulating the transcription complex of c-Jun and c-Fos, resulting in down-regulation of the transcription level of IFN-β,TNF-α,IL-1β,IL-6 and other genes controlled by the AP-1 promoter. (3) The enhancement of the binding capacity of RBM39 with PRRSV RNA hints that the improvement of viral RNA stability also contributes to the PRRSV proliferation (**Figure 9**). It is worth noting that RBM39 does not markedly affect the phosphorylation level of JNK and its upstream. In addition, the activation of the RBM39 promoter is an issue worthy of discussion.

The RNA-binding protein family can promote or inhibit the proliferation of viruses, and the mechanism of action is different. For example, Pseudouridine Synthase 4 (Pus4) could compete with the capsid protein (CP) to bind to plus-strand Brome Mosaic Virus (BMV) RNAs and prevent virion formation (38). EAP (EBER-associated protein) is a cellular RNA-binding protein which recognizes conserved stem-loop structure in the Epstein-Barr virus (EBV) small RNA EBER 1 and herpesvirus papio (HVP) small RNA homologous to EBER 1, and this may be related to regulating viral gene expression, replication and proliferation (39–42). Fátyol Károly et al. proposed the opinion that dsRNA-binding protein 2 (DRB2) of Nicotiana benthamiana plays a direct role in potato virus X (PVX)-elicited systemic necrosis through adaptive RNA interference (RNAi) pathway and innate pattern-triggered immune (PTI) responses, thereby participating in antiviral defense and restricting viral infections (8). Numerous double-stranded RNA binding proteins of plants and mammals, such as Arabidopsis DRB3, Drosophila R2D2, mammalian TRBP and PACT, are related to antiviral defense, and the mechanisms are different, which is worthy of further investigation (8, 11, 43–47).

In addition, the virus itself also encodes RNA-binding protein for its pivotal role in replication, transcription, etc. The NP protein (nucleoprotein) of influenza virus is a structural single-stranded RNA binding protein, which is a key adaptor molecule between the virus and the host cell process (48, 49). NP not only interacts with cellular polypeptides, but also interacts with various viruses and cellular macromolecules, including RNA, viral matrix proteins and viral RNA-dependent RNA polymerase, which are related to the transcription, replication, and intracellular trafficking of NP in the viral genome (48, 49). JC, Watson et al. identified a vaccinia virus-encoded double-stranded RNA-binding protein, which may be involved in inhibition of the double-stranded RNA-dependent protein kinase induced by interferon (50) and antagonized cellular antiviral response pathways triggered by dsRNA (51).

After PRRSV infection, both RBM39 and c-Jun showed the phenomenon of translocation from the nucleus to the cytoplasm. Quantities of previous studies have shown that RNA-binding proteins have been transferred from the inside to the outside of the nucleus. Another RBP protein in the RBM family, RBM14, was relocated to the nucleolus after influenza A infection (52). CIRP is a cold-induced RNA binding protein, usually located in the nucleus; however, it will migrate into the cytoplasm under a variety of physiological or stress conditions (53). For example, when human colon cancer RKO cells are irradiated by ultraviolet light, the induced genotoxic stress causes CIRP to migrate from the nucleus to the cytoplasm (54). CIRP exerts a protective effect on cells by combining specific target genes RPA2 and TRX to enhance the stability of mRNA and the translation of specific target genes (54). When the methyltransferase I at the arginine terminal in Xenopus oocytes is overexpressed, CIRP in the nucleus will transfer to the cytoplasm and accumulate (55). Under oxidative stress conditions, vascular endothelial cells will cause the CIRP in the nucleus to undergo nuclear-cytoplasmic traversal and aggregate to the stress particles in the cytoplasm (56). When salmon is under hyperosmotic stress, it will induce the production of CIRP homologue SGRP, which has the function of nuclear-cytoplasmic trafficking, allowing it to migrate into the cytoplasm (57). As predicted by the online website http://www.cbs.dtu.dk/services/NetNES/, neither RBM39 nor c-Jun has NES (leucine-rich nuclear export signals) sequences. Therefore, after PRRSV infection, the translocation of them to the cytoplasm may involve other proteins containing the NES sequence, which leading to the translocation of the two.

In higher eukaryotes, the production and expression of mature gene mRNA is a complex process that includes initiation of transcription, 5'-capping, splicing, and polyadenylation (58). RNA processing events play a vital role for specific transcription factors in pre-mRNA processing (59). Research have shown that RBM39 can regulated pre-mRNA processing in a steroid hormone receptor-dependent manner (58) and knockdown of RBM39 *via* siRNA changes the splice form of vascular endothelial growth factor A (VEGF-A) (58, 60, 61). Therefore, the mechanism of the precursor RNA splicing function of RBM39 and other RNA binding proteins after virus infection is worth exploring.

## Data Availability Statement

The raw data supporting the conclusions of this article will be made available by the authors, without undue reservation.

## Author Contributions

Conceived and designed the experiments: JH. Performed the experiments: YS, XL, YG, MZ, RS, JS, ZT, and LZ. Analyzed the data: YS and JH. Contributed reagents/materials: JH. Wrote the paper: YS and JH. All authors contributed to the article and approved the submitted version.

## Funding

This work was supported by the National Key Research and Development Program of China (2018YFD0500500) and the key underprop project of Tianjin Science and Technology Bureau in China (20YFZCSN00340).

## Conflict of Interest

The authors declare that the research was conducted in the absence of any commercial or financial relationships that could be construed as a potential conflict of interest.
