# Supplementary material for: RBM39 Alters Phosphorylation of c-Jun and Binds to Viral RNA to Promote PRRSV Proliferation
Source: Front Immunol. 2021 May 17;12:664417. doi: 10.3389/fimmu.2021.664417 (PMC8165236; doi:10.3389/fimmu.2021.664417)
Supplement: Supplementary file 1 [file DataSheet_1.pdf]

Supplementary Material

Figure S1

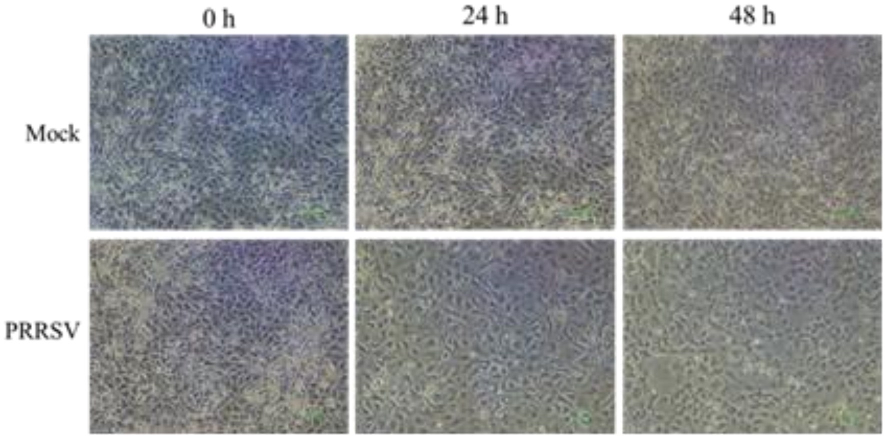

Figure S1. PRRSV infected 3D4/21 cells

Figure S2

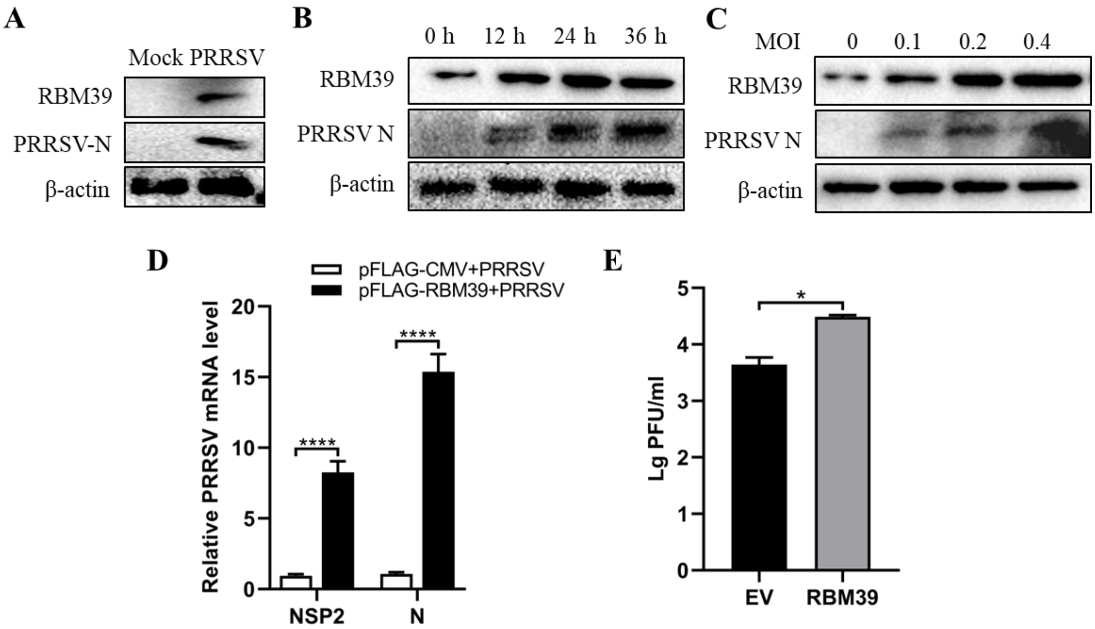

Figure S2. RBM39 contributes to PRRSV proliferation. **(A-C)** RBM39 and PRRSV protein expression levels were examined by Western blot. **(A)** Cell lysates were collected at 24 h post-infection and subjected to Western blot to analyze the protein expression. **(B)** PAM cells were infected with 0.4 MOI PRRSV and collected at the indicated times (0 h, 12 h, 24 h, 36 h) post-infection. **(C)** PAM cells were infected with different MOI (0, 0.1 0.2, 0.4) PRRSV and collected at the 24 h post-infection. **(D)** PAM cells were transfected with Flag-RBM39 plasmid or

EV (empty vetor) and infected with 0.4 MOI PRRSV. The mRNA loads of PRRSV N and nsp2 were detected by qRT-PCR. **(E)** Detection of viral titers of PRRSV in cell supernatants of RBM39 overexpressed samples. Data are representative of results from three independent experiments.

Table S1

Table S1 Transcriptome data of RBM39 after PRRSV infection

Gene Symbol	Mock count	PRRSV count	Mock fpkm	PRRSV fpkm	Log2（FC）	Products Length
RBM39	7.13	158.1	0.22	5.16	4.551795637	1593
